# Editorial: Imaging-based methods for fracture risk assessment

**DOI:** 10.3389/fendo.2025.1668924

**Published:** 2025-08-06

**Authors:** Egon Burian, Nico Sollmann, Julio Carballido-Gamio

**Affiliations:** ^1^ Department of Diagnostic and Interventional Radiology, University Hospital of Zurich, Zurich, Switzerland; ^2^ Faculty of Medicine, University of Zurich, Zurich, Switzerland; ^3^ Department of Diagnostic and Interventional Radiology, University Hospital Ulm, Ulm, Germany; ^4^ Department of Nuclear Medicine, University Hospital Ulm, Ulm, Germany; ^5^ Department of Diagnostic and Interventional Neuroradiology, School of Medicine and Health, Technical University of Munich (TUM) Klinikum Rechts der Isar, Technical University of Munich, Munich, Germany; ^6^ Technical University of Munich (TUM)-Neuroimaging Center, TUM Klinikum Rechts der Isar, Technical University of Munich, Munich, Germany; ^7^ Department of Radiology, University of Colorado, Aurora, CO, United States

**Keywords:** fracture, imaging - computed tomography, bone mineral density, MRI, proton density fat fraction

Fragility fractures represent a significant global health burden and can arise from osteoporosis (1). While areal bone mineral density (aBMD) derived from dual-energy X-ray absorptiometry (DXA) has long been the diagnostic standard, approximately 50% of fractures occur in individuals with aBMD above the osteoporotic threshold, underscoring limitations of DXA-derived aBMD in capturing the multifaceted nature of bone fragility (2–5). This necessitates a shift towards comprehensive imaging techniques and/or parameters that offer deeper insights into bone quality such as microarchitecture and biomechanical factors. This Research Topic explored recent advancements, persistent challenges, and future directions in this evolving field.

Conventional DXA provides a two-dimensional (2D) projection, simplifying bone geometry and material properties, but with measurements confounded by bone size (2). However, extracting sophisticated structural information from existing DXA scans could enhance its utility. The “Minimal Model” (MM) of hip structure, derived from DXA Hip Structural Analysis (HSA) variables like Femoral Neck Width (FNW), Sigma, and Delta, offers crucial biomechanical insights beyond simple aBMD. Zhao et al. demonstrated that the MM significantly improved hip fracture discrimination (area under the curve [AUC] = 0.838) compared to BMD alone (AUC = 0.781) in Chinese adults, with increases in FNW, Sigma, and Delta independently associated with higher hip fracture risk. This study indicated that detailed structural geometry, even from 2D imaging, might provide superior predictive power.

Quantitative computed tomography (QCT) offers three-dimensional (3D) volumetric imaging, enabling separate analysis of cortical and trabecular bone and measuring volumetric BMD (vBMD) without projection errors (2, 4). Furthermore, QCT enables assessments of bone shape and cortical bone thickness, and estimates of bone strength through finite element analysis (FEA) (6). However, the spatial resolution of QCT scans at the proximal femur and spine is not enough to visualize trabecular bone microarchitecture and its higher radiation dose and required FEA expertise limit its routine clinical use. Leveraging routine CT scans, Hounsfield Units (HU) are emerging as a valuable metric for osteoporosis assessments. Chen et al. showed that anterior column HU values were significantly lower in osteoporotic vertebral compression fractures (OVCFs) and correlated strongly with DXA T-scores (r = 0.643) and aBMD (r = 0.656). Crucially, anterior column HU demonstrated the highest correlation with vertebral compression degrees (r=0.727) and superior predictive ability for severe OVCFs (grade 3), with an optimal cutoff of 59.07 HU (AUC = 0.913). This opportunistic approach provided a localized bone quality assessment that could directly inform clinical decisions like short-term absolute bed rest post-fracture.

Chemical shift encoding-based water-fat separation MRI (CSE-MRI) is a non-invasive, radiation-free tool for assessing bone and muscle composition (5, 7, 8). Stohldreier et al. investigated 6-month changes in proton density fat fraction (PDFF) and T2* of paraspinal muscles (PSM) and vertebral bone marrow (VBM; T11-L4) as predictive biomarkers for incidental VCFs. They found that PDFF significantly increased in both PSM and VBM in patients who subsequently developed VCFs, even when opportunistic CT-based BMD remained unchanged. This suggests fatty degeneration is a crucial, early biomarker for bone fragility, challenging sole reliance on BMD Decreasing PSM T2* was also identified as a risk factor. This innovative MRI application shifts assessment from purely structural to detecting metabolic and compositional changes, potentially enabling earlier risk stratification.

Image-based biomechanical approaches, particularly FEA, aim to directly assess bone strength by simulating fracture-inducing forces. FE models, constructed from DXA or QCT data, estimate stress and strain distributions via computational modeling to predict bone strength and fracture loads (9, 10). In this regard, DXA-based FEA is accessible and comes at low radiation exposure, but relies on simplified 2D geometry. QCT-based FEA offers detailed 3D insights but incurs higher costs and radiation as well as expertise. Despite theoretical superiority, biomechanical models still face significant barriers to clinical integration. Key challenges include: (1) accurate material property characterization: current imaging struggles to quantify non-mineral components (collagen, water) crucial for bone toughness and viscoelasticity, leading to incomplete material data for FE models (Luo et al.); (2) modeling bone anisotropy: most FE models use simplified isotropic assumptions, overlooking bone’s directional strength variations, which limits accuracy in simulating multi-directional fall forces (Luo et al.); and (3) realistic fall simulations: real-world falls are unpredictable, influenced by random triggers and complex individual-specific muscle reflexes that are difficult to accurately replicate in simulations. These fundamental scientific and engineering hurdles necessitate continued research in advanced imaging, material science, and computational modeling for FEA to reach its full clinical potential.

Fracture risk extends beyond bone integrity, involving soft tissues and systemic health. Ahmed Mohamed et al. conducted a meta-analysis that revealed that preoperative thoracolumbar fascia injury (TLFI), diagnosed by MRI, may be a frequently overlooked complication in OVCFs (28% incidence) that significantly increases residual back pain post-percutaneous vertebral augmentation (odds ratio = 4.79). This highlights the need for a holistic assessment, including soft tissue integrity, for comprehensive pain management.

Furthermore, bone fragility is intertwined with systemic diseases. Jin et al. demonstrated that in patients with Parkinson’s disease (PD), hip fractures mediate the association between osteoporosis and mortality, emphasizing aggressive osteoporosis and fall management in this vulnerable population. The work by Abate et al. in liver transplant patients also identified transplant-specific factors like rejection episodes and low aBMD as critical for predicting long-term bone fragility progression. Such studies advocate for a multidisciplinary approach to fracture prevention, integrating imaging with broader clinical contexts.

The field of fracture risk assessment is transforming, moving beyond BMD to embrace sophisticated imaging techniques and parameters that capture bone quality and tissue composition. Advanced techniques like DXA-based structural analysis, localized CT-based HU measurements with opportunistic vBMD assessments, and MRI-derived biomarkers have potential to enhance predictive accuracy. The development of personalized nomograms for specific patient cohorts further exemplifies a shift towards tailored interventions. However, considerable challenges persist, including the need for robust validation in larger and diverse populations, standardization of imaging protocols, and improved computational efficiency for complex biomechanical models. Future research must prioritize hybrid and multi-modal approaches, leveraging artificial intelligence and machine learning for automated analysis and seamless clinical integration. The ultimate goal is to develop accurate, generalizable, reproducible, and user-friendly tools that can be seamlessly integrated into routine clinical workflows, fostering a holistic vision for musculoskeletal health and leading to more effective and personalized fracture prevention strategies worldwide.
